# 6-Amino-2,5-bis­(pivaloylamino)pyrimidin-4(3*H*)-one dihydrate

**DOI:** 10.1107/S1600536809019461

**Published:** 2009-06-06

**Authors:** Hoong-Kun Fun, Kasthuri Balasubramani, Anita Hazra, Manas Kumar Das, Shyamaprosad Goswami

**Affiliations:** a X-ray Crystallography Unit, School of Physics, Universiti Sains Malaysia, 11800 USM, Penang, Malaysia; b Department of Chemistry, Bengal Engineering and Science University, Shibpur, Howrah 711 103, India

## Abstract

The asymmetric unit of the title compound, C_14_H_23_N_5_O_3_·2H_2_O, contains two crystallographically independent 6-amino-2,5-bis­(pivaloylamino)pyrimidin-4(3*H*)-one mol­ecules (*A* and *B*) with similar geometry and four water mol­ecules. In both independent mol­ecules, one of the amide groups is almost coplanar with the pyrimidine ring [dihedral angle of 12.85 (9) in *A* and 12.30 (10)° in *B*], whereas the other amide group is significantly twisted away from it [dihedral angle is 72.18 (7) in *A* and 71.29 (7)° in *B*]. In each independent mol­ecule, an intra­molecular N—H⋯O hydrogen bond generates an *S*(6) ring motif. Mol­ecules *A* and *B* are linked into chains along the *a* axis by N—H⋯O and C—H⋯O hydrogen bonds. Adjacent chains are linked into a two-dimensional network parallel to the *ac* plane by water mol­ecules *via* N—H⋯O and O—H⋯O hydrogen bonds.

## Related literature

For general background on substituted pyrimidines, see: Lednicer & Mitscher (1977 ▶); Blackburn & Gait (1996 ▶); VanAllan (1976 ▶); Goswami *et al.* (2007 ▶); Brown (1988 ▶). For bond-length data, see: Allen *et al.* (1987 ▶). For the stability of the temperature controller used in the data collection, see: Cosier & Glazer (1986 ▶). For hydrogen-bond motifs, see: Bernstein *et al.* (1995 ▶).
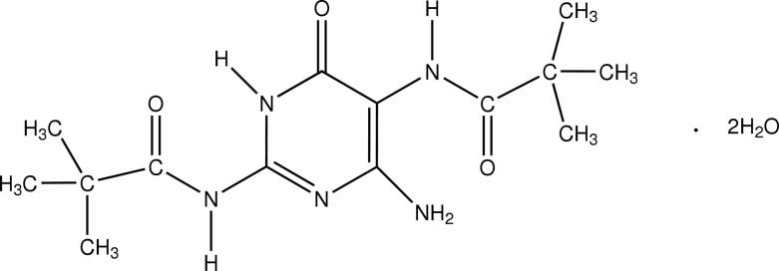


## Experimental

### 

#### Crystal data

C_14_H_23_N_5_O_3_·2H_2_O
*M* *_r_* = 345.41
Triclinic, 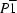

*a* = 7.5560 (3) Å
*b* = 14.1008 (6) Å
*c* = 18.0713 (6) Å
α = 71.079 (2)°
β = 89.988 (2)°
γ = 86.682 (3)°
*V* = 1817.98 (12) Å^3^
*Z* = 4
Mo *K*α radiation
μ = 0.10 mm^−1^
*T* = 100 K
0.57 × 0.19 × 0.09 mm

#### Data collection

Bruker SMART APEXII CCD area-detector diffractometer
Absorption correction: multi-scan (*SADABS*; Bruker, 2005 ▶) *T*_min_ = 0.947, *T*_max_ = 0.991
10525 measured reflections
10525 independent reflections
8199 reflections with *I* > 2σ(*I*)
*R*_int_ = 0.0000

#### Refinement

*R*[*F* ^2^ > 2σ(*F* ^2^)] = 0.063
*wR*(*F* ^2^) = 0.162
*S* = 1.11
10525 reflections
447 parameters
H-atom parameters constrained
Δρ_max_ = 0.42 e Å^−3^
Δρ_min_ = −0.35 e Å^−3^

### 

Data collection: *APEX2* (Bruker, 2005 ▶); cell refinement: *SAINT* (Bruker, 2005 ▶); data reduction: *SAINT*; program(s) used to solve structure: *SHELXTL* (Sheldrick, 2008 ▶); program(s) used to refine structure: *SHELXTL*; molecular graphics: *SHELXTL*; software used to prepare material for publication: *SHELXTL* and *PLATON* (Spek, 2009 ▶).

## Supplementary Material

Crystal structure: contains datablocks global, I. DOI: 10.1107/S1600536809019461/ci2809sup1.cif

Structure factors: contains datablocks I. DOI: 10.1107/S1600536809019461/ci2809Isup2.hkl

Additional supplementary materials:  crystallographic information; 3D view; checkCIF report

## Figures and Tables

**Table 1:** Hydrogen-bond geometry (Å, °)

*D*—H⋯*A*	*D*—H	H⋯*A*	*D*⋯*A*	*D*—H⋯*A*
N4*A*—H4*AA*⋯O1*W*^i^	0.86	2.07	2.918 (2)	167
N4*B*—H4*BA*⋯O4*W*^ii^	0.86	2.08	2.920 (2)	166
N5*B*—H5*BA*⋯O4*W*^iii^	0.86	2.32	3.160 (2)	166
N5*B*—H5*BB*⋯O1*A*^iv^	0.86	2.09	2.861 (2)	149
O1*W*—H1*W*1⋯O2*W*^v^	0.87	2.00	2.857 (2)	167
O1*W*—H2*W*1⋯O2*W*^vi^	0.90	1.92	2.819 (2)	178
O2*W*—H2*W*2⋯O2*B*^v^	0.89	1.96	2.824 (2)	162
O3*W*—H1*W*3⋯O2*A*^iii^	0.86	1.91	2.722 (2)	158
O3*W*—H2*W*3⋯O2*A*^vii^	0.89	1.97	2.833 (2)	162
O4*W*—H1*W*4⋯O3*W*^viii^	0.88	1.99	2.865 (2)	174
N3*A*—H3*AA*⋯O3*A*	0.86	1.98	2.633 (2)	132
N5*A*—H5*AA*⋯O1*W*	0.86	2.32	3.163 (2)	167
N5*A*—H5*AB*⋯O1*B*	0.86	2.08	2.854 (2)	149
N3*B*—H3*BA*⋯O3*B*	0.86	1.97	2.632 (2)	132
O2*W*—H1*W*2⋯O2*B*	0.87	1.91	2.717 (2)	154
O4*W*—H2*W*4⋯O3*W*	0.88	1.93	2.811 (2)	173
C14*A*—H14*A*⋯O1*B*	0.96	2.53	3.355 (3)	144

Symmetry codes: (i) 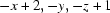; (ii) 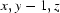; (iii) 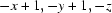; (iv) 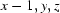; (v) 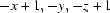; (vi) 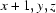; (vii) 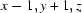; (viii) 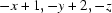.

**Fig. 1.**
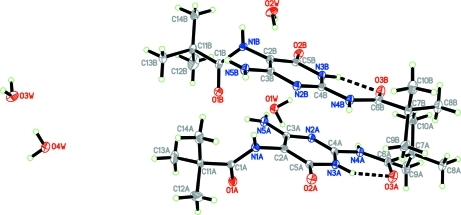
The asymmetric unit of the title compound, showing 50% probability displacement ellipsoids and the atom-numbering scheme. Dashed lines indicate hydrogen bonding.

**Fig. 2.**
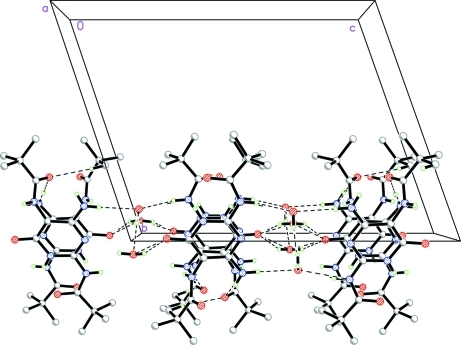
Part of the crystal packing of the title compound, viewed down the a axis. Dashed lines indicate hydrogen bonding.

## supplementary crystallographic information

### Comment

Various drugs and biologically active molecules contain substituted pyrimidines (Lednicer & Mitscher, 1977). Adenine, uracil, thyamine are pyrimidine-based bases in nucleic acids (Blackburn & Gait, 1996). 2 5,6-Triamino-3*H*- pyrimidin-4-one dihydrochloride (VanAllan, 1976; Goswami *et al.* 2007) is an important component for the synthesis of pterin molecules (Brown, 1988). The title compound was selectively synthesized by the reaction of 2,5,6-triamino-3*H*-pyrimidin-4-one dihydrochloride with pivalic anhydride and its crystal structure is reported here.

There are two crystallographically independent 6-amino-2,5-dipivaloyl-3*H*-pyrimidin-4-one molecules, A and B, and four water molecules in the asymmetric unit of the title compound (Fig 1). Molecules A and B have similar geometry. The bond lengths (Allen *et al.*, 1987) and angles are normal. In both A and B, one of the amide groups is almost coplanar with the pyrimidine ring (dihedral angle is 12.85 (9)° in A and 12.30 (10)° in B) whereas the other is significantly twisted away from the pyrimidine ring (dihedral angle is 72.18 (7)° in A and 71.29 (07)° in B) In each independent molecule, an intramolecular N—H···O hydrogen bond generates an S(6) ring motif (Bernstein *et al.*, 1995).

The independent molecules are linked into chains along the *a* axis by N—H···O and C—H···O hydrogen bonds. The adjacent chains are linked into a two-dimensional network parallel to the *ac* plane (Fig.2) by water molecules via N—H···O and O—H···O hydrogen bonds (Table 1).

### Experimental

2,5,6-Triaminopyrimidine-4-(3*H*)-one dihydrochloride (200mg, 0.93mmol) was heated with pivalic anhydride (1 ml) at 393 K for 6 h in the presence of a catalytic amount of 4-dimethylaminopyridine (DMAP) (10 mol%). After the formation of a major amount of dipivaloyl product as monitored by TLC, the solid residue was washed with petroleum ether to remove the excess pivalic anhydride. The solid residue was purified through silica gel (100–200 mesh) column chromatography eluting 3% methanol in chloroform to get the pure crystalline solid. Single crystals were grown by slow evaporation of a chloroform solution (m.p. 523-525 K). IR: 3416, 3217, 2965, 2873,1645, 1568, 1488, 1438, 1240, 1176, 763 cm^-1^. ^1^H NMR (CDCl_3_, 400 MHz): δ(p.p.m.): 11.61 (bs, 1H), 8.27 (bs, 1H), 7.64 (bs, 1H), 5.35 (bs, 2H), 1.28 (s, 9H), 1.24 (s, 9H). LC—MS: m/*z* (%): 310.4[(*M*+H)^+^,40], 292.3 (100), 186.3.

### Refinement

H atoms were positioned geometrically (N-H = 0.86 Å and C-H = 0.93–0.96 Å) and refined using a riding model with *U*_iso_(H) = 1.2*U*_eq_(C,N) and 1.5*U*_eq_(methyl C). A rotating–group model was used for the methyl groups. The H atoms of the water molecules were located in a difference Fourier map and constrained to ride on their parent atom, with *U*_iso_(H) = 1.5*U*_eq_(O). The crystal was a pseudo-merohedral triplet with ratio 0.764 (5):0.155 (5):0.081 (5). The refined  BASF parameters are 0.155 (5) and 0.081 (5).

### Crystal data

**Table:**

C_14_H_23_N_5_O_3_·2H_2_O	*Z* = 4
*M**_r_* = 345.41	*F*(000) = 744
Triclinic, *P*1	*D*_x_ = 1.262 Mg m^−^^3^
Hall symbol: -P 1	Mo *K*α radiation, λ = 0.71073 Å
*a* = 7.5560 (3) Å	Cell parameters from 8925 reflections
*b* = 14.1008 (6) Å	θ = 3.1–32.5°
*c* = 18.0713 (6) Å	µ = 0.10 mm^−^^1^
α = 71.079 (2)°	*T* = 100 K
β = 89.988 (2)°	Block, colourless
γ = 86.682 (3)°	0.57 × 0.19 × 0.09 mm
*V* = 1817.98 (12) Å^3^	

### Data collection

**Table:**

Bruker SMART APEXII CCD area-detector diffractometer	10525 independent reflections
Radiation source: fine-focus sealed tube	8199 reflections with *I* > 2σ(*I*)
graphite	*R*_int_ = 0.0000
φ and ω scans	θ_max_ = 30.0°, θ_min_ = 1.2°
Absorption correction: multi-scan (*SADABS*; Bruker, 2005)	*h* = −10→10
*T*_min_ = 0.947, *T*_max_ = 0.991	*k* = −18→19
10525 measured reflections	*l* = 0→25

### Refinement

**Table:**

Refinement on *F*^2^	Primary atom site location: structure-invariant direct methods
Least-squares matrix: full	Secondary atom site location: difference Fourier map
*R*[*F*^2^ > 2σ(*F*^2^)] = 0.063	Hydrogen site location: inferred from neighbouring sites
*wR*(*F*^2^) = 0.162	H-atom parameters constrained
*S* = 1.11	*w* = 1/[σ^2^(*F*_o_^2^) + (0.0332*P*)^2^ + 1.7967*P*] where *P* = (*F*_o_^2^ + 2*F*_c_^2^)/3
10525 reflections	(Δ/σ)_max_ = 0.001
447 parameters	Δρ_max_ = 0.42 e Å^−^^3^
0 restraints	Δρ_min_ = −0.35 e Å^−^^3^

### Special details

**Table:**

Experimental. The crystal was placed in the cold stream of an Oxford Cyrosystems Cobra open-flow nitrogen cryostat (Cosier & Glazer, 1986) operating at 100.0 (1) K.
Geometry. All e.s.d.'s (except the e.s.d. in the dihedral angle between two l.s. planes) are estimated using the full covariance matrix. The cell e.s.d.'s are taken into account individually in the estimation of e.s.d.'s in distances, angles and torsion angles; correlations between e.s.d.'s in cell parameters are only used when they are defined by crystal symmetry. An approximate (isotropic) treatment of cell e.s.d.'s is used for estimating e.s.d.'s involving l.s. planes.
Refinement. Refinement of *F*^2^ against ALL reflections. The weighted *R*-factor *wR* and goodness of fit *S* are based on *F*^2^, conventional *R*-factors *R* are based on *F*, with *F* set to zero for negative *F*^2^. The threshold expression of *F*^2^ > σ(*F*^2^) is used only for calculating *R*-factors(gt) *etc*. and is not relevant to the choice of reflections for refinement. *R*-factors based on *F*^2^ are statistically about twice as large as those based on *F*, and *R*- factors based on ALL data will be even larger.

### Fractional atomic coordinates and isotropic or equivalent isotropic displacement parameters (Å^2^)

**Table:**

	*x*	*y*	*z*	*U*_iso_*/*U*_eq_	
O1A	1.0113 (2)	0.26217 (11)	0.14678 (9)	0.0172 (3)	
O2A	0.9781 (2)	−0.00932 (11)	0.11107 (8)	0.0163 (3)	
O3A	1.2104 (2)	−0.26095 (12)	0.29920 (9)	0.0211 (3)	
N1A	0.7981 (2)	0.15883 (12)	0.14173 (9)	0.0126 (3)	
H1AA	0.6995	0.1522	0.1207	0.015*	
N2A	0.9705 (2)	−0.00908 (12)	0.33668 (9)	0.0124 (3)	
N3A	1.0445 (2)	−0.08254 (12)	0.24031 (9)	0.0126 (3)	
H3AA	1.0950	−0.1334	0.2306	0.015*	
N4A	1.1220 (2)	−0.16358 (12)	0.37291 (9)	0.0129 (3)	
H4AA	1.1299	−0.1587	0.4190	0.016*	
N5A	0.8070 (2)	0.14048 (13)	0.30396 (10)	0.0151 (3)	
H5AA	0.8136	0.1356	0.3526	0.018*	
H5AB	0.7506	0.1916	0.2712	0.018*	
C1A	0.8701 (3)	0.24963 (15)	0.11802 (11)	0.0121 (4)	
C2A	0.8808 (3)	0.07412 (14)	0.20033 (11)	0.0121 (4)	
C3A	0.8843 (3)	0.06879 (14)	0.27938 (11)	0.0120 (4)	
C4A	1.0438 (3)	−0.08141 (14)	0.31492 (11)	0.0126 (4)	
C5A	0.9655 (3)	−0.00353 (15)	0.17828 (11)	0.0120 (4)	
C6A	1.1882 (3)	−0.25228 (15)	0.36395 (11)	0.0137 (4)	
C7A	1.2277 (3)	−0.33855 (15)	0.44031 (12)	0.0154 (4)	
C8A	1.2899 (3)	−0.43211 (17)	0.42020 (13)	0.0211 (5)	
H8AA	1.1995	−0.4482	0.3896	0.032*	
H8AB	1.3124	−0.4874	0.4676	0.032*	
H8AC	1.3967	−0.4196	0.3907	0.032*	
C9A	1.3758 (4)	−0.31103 (18)	0.48672 (14)	0.0228 (5)	
H9AA	1.3385	−0.2512	0.4983	0.034*	
H9AB	1.4811	−0.2995	0.4561	0.034*	
H9AC	1.4001	−0.3652	0.5347	0.034*	
C10A	1.0576 (3)	−0.35827 (17)	0.48821 (13)	0.0217 (5)	
H10A	0.9629	−0.3673	0.4562	0.033*	
H10B	1.0265	−0.3021	0.5056	0.033*	
H10C	1.0771	−0.4178	0.5328	0.033*	
C11A	0.7768 (3)	0.33580 (15)	0.05214 (12)	0.0152 (4)	
C12A	0.9017 (4)	0.3536 (2)	−0.01777 (14)	0.0334 (6)	
H12A	0.8555	0.4103	−0.0604	0.050*	
H12B	0.9102	0.2951	−0.0340	0.050*	
H12C	1.0172	0.3666	−0.0026	0.050*	
C13A	0.7607 (4)	0.42818 (17)	0.07890 (17)	0.0278 (5)	
H13A	0.6818	0.4161	0.1222	0.042*	
H13B	0.7147	0.4852	0.0365	0.042*	
H13C	0.8754	0.4413	0.0948	0.042*	
C14A	0.5943 (3)	0.31374 (17)	0.02795 (14)	0.0226 (5)	
H14A	0.5179	0.2988	0.0722	0.034*	
H14B	0.6054	0.2572	0.0093	0.034*	
H14C	0.5447	0.3714	−0.0129	0.034*	
O1B	0.5105 (2)	0.26137 (11)	0.21892 (9)	0.0177 (3)	
O2B	0.4795 (2)	−0.01130 (11)	0.39406 (8)	0.0160 (3)	
O3B	0.7098 (2)	−0.26303 (12)	0.33417 (9)	0.0213 (3)	
N1B	0.3005 (2)	0.15684 (12)	0.27823 (10)	0.0124 (3)	
H1BA	0.2027	0.1501	0.3032	0.015*	
N2B	0.4710 (2)	−0.01103 (12)	0.16825 (9)	0.0124 (3)	
N3B	0.5460 (2)	−0.08400 (12)	0.30196 (9)	0.0125 (3)	
H3BA	0.5972	−0.1345	0.3374	0.015*	
N4B	0.6226 (2)	−0.16539 (13)	0.21065 (10)	0.0135 (3)	
H4BA	0.6310	−0.1607	0.1622	0.016*	
N5B	0.3072 (2)	0.13860 (13)	0.12500 (10)	0.0143 (3)	
H5BA	0.3129	0.1333	0.0790	0.017*	
H5BB	0.2512	0.1899	0.1319	0.017*	
C1B	0.3719 (3)	0.24772 (15)	0.25544 (11)	0.0123 (4)	
C2B	0.3819 (3)	0.07227 (14)	0.26221 (11)	0.0124 (4)	
C3B	0.3855 (3)	0.06723 (14)	0.18588 (11)	0.0113 (3)	
C4B	0.5443 (3)	−0.08344 (14)	0.22681 (11)	0.0118 (4)	
C5B	0.4673 (3)	−0.00523 (15)	0.32382 (11)	0.0129 (4)	
C6B	0.6886 (3)	−0.25413 (15)	0.26499 (12)	0.0149 (4)	
C7B	0.7303 (3)	−0.34013 (16)	0.23251 (12)	0.0162 (4)	
C8B	0.7917 (4)	−0.43431 (17)	0.30020 (14)	0.0226 (5)	
H8BA	0.7000	−0.4511	0.3382	0.034*	
H8BB	0.8970	−0.4216	0.3242	0.034*	
H8BC	0.8165	−0.4892	0.2805	0.034*	
C9B	0.8798 (4)	−0.31215 (18)	0.17286 (14)	0.0232 (5)	
H9BA	0.8418	−0.2534	0.1302	0.035*	
H9BB	0.9078	−0.3669	0.1534	0.035*	
H9BC	0.9832	−0.2987	0.1978	0.035*	
C10B	0.5631 (3)	−0.36058 (17)	0.19338 (14)	0.0231 (5)	
H10D	0.5312	−0.3038	0.1481	0.035*	
H10E	0.4676	−0.3716	0.2297	0.035*	
H10F	0.5858	−0.4191	0.1779	0.035*	
C11B	0.2801 (3)	0.33333 (16)	0.27831 (12)	0.0150 (4)	
C12B	0.4114 (4)	0.3547 (2)	0.33506 (17)	0.0323 (6)	
H12D	0.4266	0.2970	0.3813	0.049*	
H12E	0.3659	0.4114	0.3491	0.049*	
H12F	0.5235	0.3691	0.3102	0.049*	
C13B	0.2533 (3)	0.42518 (17)	0.20411 (14)	0.0214 (5)	
H13D	0.1719	0.4108	0.1689	0.032*	
H13E	0.3650	0.4404	0.1791	0.032*	
H13F	0.2062	0.4817	0.2180	0.032*	
C14B	0.1021 (4)	0.30911 (17)	0.31798 (15)	0.0236 (5)	
H14D	0.0220	0.2929	0.2833	0.035*	
H14E	0.0533	0.3663	0.3305	0.035*	
H14F	0.1191	0.2529	0.3651	0.035*	
O1W	0.8987 (2)	0.12286 (11)	0.47888 (8)	0.0179 (3)	
H1W1	0.8648	0.0614	0.4942	0.027*	
H2W1	1.0149	0.1036	0.4798	0.027*	
O2W	0.2630 (2)	0.06473 (13)	0.48386 (9)	0.0219 (3)	
H1W2	0.3371	0.0602	0.4483	0.033*	
H2W2	0.3376	0.0610	0.5230	0.033*	
O3W	0.2385 (2)	0.93480 (13)	0.01760 (9)	0.0225 (3)	
H1W3	0.1642	0.9417	−0.0197	0.034*	
H2W3	0.1705	0.9430	0.0559	0.034*	
O4W	0.6016 (2)	0.87651 (11)	0.04160 (9)	0.0181 (3)	
H1W4	0.6432	0.9365	0.0213	0.027*	
H2W4	0.4873	0.8911	0.0321	0.027*	

### Atomic displacement parameters (Å^2^)

**Table:**

	*U*^11^	*U*^22^	*U*^33^	*U*^12^	*U*^13^	*U*^23^
O1A	0.0161 (7)	0.0141 (7)	0.0198 (7)	−0.0005 (6)	−0.0036 (6)	−0.0033 (6)
O2A	0.0219 (8)	0.0187 (7)	0.0083 (6)	0.0010 (6)	−0.0016 (5)	−0.0050 (5)
O3A	0.0304 (9)	0.0205 (8)	0.0122 (7)	0.0059 (7)	−0.0003 (6)	−0.0064 (6)
N1A	0.0131 (8)	0.0133 (8)	0.0096 (7)	−0.0008 (6)	−0.0027 (6)	−0.0013 (6)
N2A	0.0155 (8)	0.0121 (8)	0.0091 (7)	0.0010 (6)	−0.0006 (6)	−0.0032 (6)
N3A	0.0166 (9)	0.0113 (7)	0.0098 (7)	0.0017 (6)	0.0001 (6)	−0.0038 (6)
N4A	0.0181 (9)	0.0128 (8)	0.0076 (7)	0.0011 (6)	−0.0022 (6)	−0.0034 (6)
N5A	0.0211 (9)	0.0140 (8)	0.0099 (7)	0.0028 (7)	−0.0001 (6)	−0.0039 (6)
C1A	0.0135 (9)	0.0124 (9)	0.0104 (8)	0.0001 (7)	0.0030 (7)	−0.0039 (7)
C2A	0.0139 (9)	0.0117 (8)	0.0093 (8)	−0.0004 (7)	−0.0003 (7)	−0.0016 (6)
C3A	0.0122 (9)	0.0121 (8)	0.0115 (8)	−0.0020 (7)	0.0008 (7)	−0.0034 (7)
C4A	0.0144 (9)	0.0121 (9)	0.0101 (8)	−0.0021 (7)	−0.0001 (7)	−0.0018 (7)
C5A	0.0130 (9)	0.0123 (9)	0.0100 (8)	−0.0011 (7)	−0.0006 (7)	−0.0025 (7)
C6A	0.0161 (10)	0.0127 (9)	0.0117 (8)	0.0008 (7)	−0.0024 (7)	−0.0036 (7)
C7A	0.0212 (11)	0.0116 (9)	0.0123 (8)	0.0031 (8)	−0.0009 (7)	−0.0028 (7)
C8A	0.0267 (12)	0.0162 (10)	0.0195 (10)	0.0064 (9)	−0.0036 (9)	−0.0060 (8)
C9A	0.0289 (13)	0.0181 (10)	0.0203 (10)	0.0041 (9)	−0.0108 (9)	−0.0058 (8)
C10A	0.0285 (12)	0.0152 (10)	0.0176 (10)	0.0022 (9)	0.0055 (9)	−0.0008 (8)
C11A	0.0162 (10)	0.0114 (9)	0.0134 (9)	0.0014 (7)	0.0011 (7)	0.0020 (7)
C12A	0.0294 (14)	0.0379 (14)	0.0190 (11)	0.0033 (11)	0.0085 (10)	0.0091 (10)
C13A	0.0262 (13)	0.0136 (10)	0.0416 (14)	0.0029 (9)	−0.0079 (11)	−0.0068 (10)
C14A	0.0252 (12)	0.0180 (10)	0.0197 (10)	0.0009 (9)	−0.0074 (9)	0.0003 (8)
O1B	0.0167 (8)	0.0150 (7)	0.0211 (7)	−0.0010 (6)	0.0034 (6)	−0.0054 (6)
O2B	0.0196 (8)	0.0199 (7)	0.0100 (6)	0.0030 (6)	−0.0009 (5)	−0.0078 (5)
O3B	0.0317 (9)	0.0179 (7)	0.0140 (7)	0.0072 (6)	−0.0030 (6)	−0.0061 (6)
N1B	0.0126 (8)	0.0126 (8)	0.0144 (7)	0.0003 (6)	0.0023 (6)	−0.0077 (6)
N2B	0.0147 (8)	0.0128 (8)	0.0109 (7)	0.0001 (6)	0.0002 (6)	−0.0057 (6)
N3B	0.0163 (9)	0.0116 (7)	0.0096 (7)	0.0020 (6)	−0.0005 (6)	−0.0039 (6)
N4B	0.0189 (9)	0.0134 (8)	0.0099 (7)	0.0019 (7)	0.0003 (6)	−0.0065 (6)
N5B	0.0188 (9)	0.0136 (8)	0.0112 (7)	0.0028 (6)	−0.0018 (6)	−0.0055 (6)
C1B	0.0147 (10)	0.0115 (9)	0.0109 (8)	0.0016 (7)	−0.0033 (7)	−0.0045 (7)
C2B	0.0141 (9)	0.0123 (9)	0.0131 (8)	−0.0008 (7)	0.0005 (7)	−0.0075 (7)
C3B	0.0111 (9)	0.0118 (8)	0.0115 (8)	−0.0015 (7)	−0.0005 (7)	−0.0046 (7)
C4B	0.0120 (9)	0.0120 (8)	0.0128 (8)	−0.0008 (7)	0.0012 (7)	−0.0060 (7)
C5B	0.0136 (9)	0.0144 (9)	0.0128 (8)	−0.0017 (7)	0.0026 (7)	−0.0072 (7)
C6B	0.0149 (10)	0.0137 (9)	0.0167 (9)	0.0006 (7)	0.0012 (7)	−0.0061 (7)
C7B	0.0213 (11)	0.0139 (9)	0.0151 (9)	0.0025 (8)	−0.0003 (8)	−0.0077 (7)
C8B	0.0314 (13)	0.0146 (10)	0.0207 (10)	0.0058 (9)	−0.0015 (9)	−0.0052 (8)
C9B	0.0279 (13)	0.0197 (11)	0.0213 (10)	0.0055 (9)	0.0072 (9)	−0.0070 (8)
C10B	0.0278 (13)	0.0183 (10)	0.0254 (11)	0.0016 (9)	−0.0080 (9)	−0.0104 (9)
C11B	0.0182 (10)	0.0127 (9)	0.0156 (9)	0.0024 (8)	−0.0023 (8)	−0.0072 (7)
C12B	0.0392 (16)	0.0310 (13)	0.0356 (14)	0.0070 (11)	−0.0154 (12)	−0.0244 (11)
C13B	0.0234 (12)	0.0134 (10)	0.0259 (11)	0.0010 (8)	0.0008 (9)	−0.0048 (8)
C14B	0.0283 (13)	0.0164 (10)	0.0272 (11)	0.0030 (9)	0.0100 (10)	−0.0092 (9)
O1W	0.0225 (8)	0.0167 (7)	0.0140 (7)	0.0023 (6)	0.0011 (6)	−0.0049 (5)
O2W	0.0174 (8)	0.0347 (9)	0.0155 (7)	0.0048 (7)	0.0001 (6)	−0.0119 (6)
O3W	0.0190 (8)	0.0352 (9)	0.0148 (7)	0.0039 (7)	−0.0031 (6)	−0.0111 (6)
O4W	0.0209 (8)	0.0176 (7)	0.0162 (7)	0.0037 (6)	−0.0026 (6)	−0.0067 (6)

### Geometric parameters (Å, °)

**Table:**

O1A—C1A	1.233 (3)	N1B—C2B	1.422 (2)
O2A—C5A	1.247 (2)	N1B—H1BA	0.86
O3A—C6A	1.226 (2)	N2B—C4B	1.305 (3)
N1A—C1A	1.358 (2)	N2B—C3B	1.372 (2)
N1A—C2A	1.426 (2)	N3B—C4B	1.355 (2)
N1A—H1AA	0.86	N3B—C5B	1.397 (2)
N2A—C4A	1.302 (3)	N3B—H3BA	0.86
N2A—C3A	1.372 (3)	N4B—C6B	1.382 (3)
N3A—C4A	1.354 (2)	N4B—C4B	1.382 (2)
N3A—C5A	1.403 (2)	N4B—H4BA	0.86
N3A—H3AA	0.86	N5B—C3B	1.336 (2)
N4A—C6A	1.378 (3)	N5B—H5BA	0.86
N4A—C4A	1.387 (2)	N5B—H5BB	0.86
N4A—H4AA	0.86	C1B—C11B	1.529 (3)
N5A—C3A	1.335 (3)	C2B—C3B	1.404 (3)
N5A—H5AA	0.86	C2B—C5B	1.407 (3)
N5A—H5AB	0.86	C6B—C7B	1.527 (3)
C1A—C11A	1.534 (3)	C7B—C8B	1.533 (3)
C2A—C5A	1.403 (3)	C7B—C10B	1.535 (3)
C2A—C3A	1.406 (3)	C7B—C9B	1.539 (3)
C6A—C7A	1.531 (3)	C8B—H8BA	0.96
C7A—C8A	1.527 (3)	C8B—H8BB	0.96
C7A—C10A	1.538 (3)	C8B—H8BC	0.96
C7A—C9A	1.538 (3)	C9B—H9BA	0.96
C8A—H8AA	0.96	C9B—H9BB	0.96
C8A—H8AB	0.96	C9B—H9BC	0.96
C8A—H8AC	0.96	C10B—H10D	0.96
C9A—H9AA	0.96	C10B—H10E	0.96
C9A—H9AB	0.96	C10B—H10F	0.96
C9A—H9AC	0.96	C11B—C14B	1.527 (3)
C10A—H10A	0.96	C11B—C13B	1.536 (3)
C10A—H10B	0.96	C11B—C12B	1.537 (3)
C10A—H10C	0.96	C12B—H12D	0.96
C11A—C14A	1.526 (3)	C12B—H12E	0.96
C11A—C13A	1.528 (3)	C12B—H12F	0.96
C11A—C12A	1.539 (3)	C13B—H13D	0.96
C12A—H12A	0.96	C13B—H13E	0.96
C12A—H12B	0.96	C13B—H13F	0.96
C12A—H12C	0.96	C14B—H14D	0.96
C13A—H13A	0.96	C14B—H14E	0.96
C13A—H13B	0.96	C14B—H14F	0.96
C13A—H13C	0.96	O1W—H1W1	0.87
C14A—H14A	0.96	O1W—H2W1	0.90
C14A—H14B	0.96	O2W—H1W2	0.87
C14A—H14C	0.96	O2W—H2W2	0.89
O1B—C1B	1.229 (3)	O3W—H1W3	0.85
O2B—C5B	1.247 (2)	O3W—H2W3	0.89
O3B—C6B	1.225 (3)	O4W—H1W4	0.88
N1B—C1B	1.357 (3)	O4W—H2W4	0.88
			
C1A—N1A—C2A	122.09 (17)	C2B—N1B—H1BA	118.9
C1A—N1A—H1AA	119.0	C4B—N2B—C3B	116.57 (16)
C2A—N1A—H1AA	119.0	C4B—N3B—C5B	122.21 (17)
C4A—N2A—C3A	116.50 (16)	C4B—N3B—H3BA	118.9
C4A—N3A—C5A	122.14 (17)	C5B—N3B—H3BA	118.9
C4A—N3A—H3AA	118.9	C6B—N4B—C4B	126.21 (17)
C5A—N3A—H3AA	118.9	C6B—N4B—H4BA	116.9
C6A—N4A—C4A	126.41 (17)	C4B—N4B—H4BA	116.9
C6A—N4A—H4AA	116.8	C3B—N5B—H5BA	120.0
C4A—N4A—H4AA	116.8	C3B—N5B—H5BB	120.0
C3A—N5A—H5AA	120.0	H5BA—N5B—H5BB	120.0
C3A—N5A—H5AB	120.0	O1B—C1B—N1B	121.35 (18)
H5AA—N5A—H5AB	120.0	O1B—C1B—C11B	119.82 (18)
O1A—C1A—N1A	120.91 (18)	N1B—C1B—C11B	118.79 (18)
O1A—C1A—C11A	120.20 (18)	C3B—C2B—C5B	119.80 (17)
N1A—C1A—C11A	118.84 (18)	C3B—C2B—N1B	121.26 (17)
C5A—C2A—C3A	119.52 (17)	C5B—C2B—N1B	118.87 (17)
C5A—C2A—N1A	119.35 (17)	N5B—C3B—N2B	115.08 (17)
C3A—C2A—N1A	121.09 (17)	N5B—C3B—C2B	122.54 (18)
N5A—C3A—N2A	114.99 (17)	N2B—C3B—C2B	122.38 (17)
N5A—C3A—C2A	122.35 (18)	N2B—C4B—N3B	124.38 (18)
N2A—C3A—C2A	122.64 (18)	N2B—C4B—N4B	117.28 (17)
N2A—C4A—N3A	124.42 (18)	N3B—C4B—N4B	118.33 (17)
N2A—C4A—N4A	117.21 (17)	O2B—C5B—N3B	117.56 (18)
N3A—C4A—N4A	118.35 (17)	O2B—C5B—C2B	127.85 (18)
O2A—C5A—N3A	117.72 (18)	N3B—C5B—C2B	114.59 (17)
O2A—C5A—C2A	127.59 (18)	O3B—C6B—N4B	122.21 (18)
N3A—C5A—C2A	114.70 (17)	O3B—C6B—C7B	122.76 (19)
O3A—C6A—N4A	121.84 (18)	N4B—C6B—C7B	115.02 (17)
O3A—C6A—C7A	123.05 (18)	C6B—C7B—C8B	108.78 (17)
N4A—C6A—C7A	115.10 (17)	C6B—C7B—C10B	109.49 (19)
C8A—C7A—C6A	108.48 (17)	C8B—C7B—C10B	109.59 (19)
C8A—C7A—C10A	109.87 (19)	C6B—C7B—C9B	109.57 (18)
C6A—C7A—C10A	109.06 (18)	C8B—C7B—C9B	109.16 (19)
C8A—C7A—C9A	109.16 (19)	C10B—C7B—C9B	110.22 (19)
C6A—C7A—C9A	109.61 (18)	C7B—C8B—H8BA	109.5
C10A—C7A—C9A	110.63 (18)	C7B—C8B—H8BB	109.5
C7A—C8A—H8AA	109.5	H8BA—C8B—H8BB	109.5
C7A—C8A—H8AB	109.5	C7B—C8B—H8BC	109.5
H8AA—C8A—H8AB	109.5	H8BA—C8B—H8BC	109.5
C7A—C8A—H8AC	109.5	H8BB—C8B—H8BC	109.5
H8AA—C8A—H8AC	109.5	C7B—C9B—H9BA	109.5
H8AB—C8A—H8AC	109.5	C7B—C9B—H9BB	109.5
C7A—C9A—H9AA	109.5	H9BA—C9B—H9BB	109.5
C7A—C9A—H9AB	109.5	C7B—C9B—H9BC	109.5
H9AA—C9A—H9AB	109.5	H9BA—C9B—H9BC	109.5
C7A—C9A—H9AC	109.5	H9BB—C9B—H9BC	109.5
H9AA—C9A—H9AC	109.5	C7B—C10B—H10D	109.5
H9AB—C9A—H9AC	109.5	C7B—C10B—H10E	109.5
C7A—C10A—H10A	109.5	H10D—C10B—H10E	109.5
C7A—C10A—H10B	109.5	C7B—C10B—H10F	109.5
H10A—C10A—H10B	109.5	H10D—C10B—H10F	109.5
C7A—C10A—H10C	109.5	H10E—C10B—H10F	109.5
H10A—C10A—H10C	109.5	C14B—C11B—C1B	114.60 (18)
H10B—C10A—H10C	109.5	C14B—C11B—C13B	109.37 (19)
C14A—C11A—C13A	109.63 (19)	C1B—C11B—C13B	108.17 (17)
C14A—C11A—C1A	114.71 (17)	C14B—C11B—C12B	109.6 (2)
C13A—C11A—C1A	107.86 (18)	C1B—C11B—C12B	104.75 (18)
C14A—C11A—C12A	109.2 (2)	C13B—C11B—C12B	110.2 (2)
C13A—C11A—C12A	110.5 (2)	C11B—C12B—H12D	109.5
C1A—C11A—C12A	104.87 (18)	C11B—C12B—H12E	109.5
C11A—C12A—H12A	109.5	H12D—C12B—H12E	109.5
C11A—C12A—H12B	109.5	C11B—C12B—H12F	109.5
H12A—C12A—H12B	109.5	H12D—C12B—H12F	109.5
C11A—C12A—H12C	109.5	H12E—C12B—H12F	109.5
H12A—C12A—H12C	109.5	C11B—C13B—H13D	109.5
H12B—C12A—H12C	109.5	C11B—C13B—H13E	109.5
C11A—C13A—H13A	109.5	H13D—C13B—H13E	109.5
C11A—C13A—H13B	109.5	C11B—C13B—H13F	109.5
H13A—C13A—H13B	109.5	H13D—C13B—H13F	109.5
C11A—C13A—H13C	109.5	H13E—C13B—H13F	109.5
H13A—C13A—H13C	109.5	C11B—C14B—H14D	109.5
H13B—C13A—H13C	109.5	C11B—C14B—H14E	109.5
C11A—C14A—H14A	109.5	H14D—C14B—H14E	109.5
C11A—C14A—H14B	109.5	C11B—C14B—H14F	109.5
H14A—C14A—H14B	109.5	H14D—C14B—H14F	109.5
C11A—C14A—H14C	109.5	H14E—C14B—H14F	109.5
H14A—C14A—H14C	109.5	H1W1—O1W—H2W1	93.9
H14B—C14A—H14C	109.5	H1W2—O2W—H2W2	100.6
C1B—N1B—C2B	122.25 (17)	H1W3—O3W—H2W3	103.4
C1B—N1B—H1BA	118.9	H1W4—O4W—H2W4	100.7
			
C2A—N1A—C1A—O1A	−0.1 (3)	C2B—N1B—C1B—O1B	0.7 (3)
C2A—N1A—C1A—C11A	177.39 (17)	C2B—N1B—C1B—C11B	−177.27 (17)
C1A—N1A—C2A—C5A	−106.0 (2)	C1B—N1B—C2B—C3B	−70.7 (3)
C1A—N1A—C2A—C3A	71.8 (3)	C1B—N1B—C2B—C5B	106.3 (2)
C4A—N2A—C3A—N5A	178.46 (18)	C4B—N2B—C3B—N5B	−178.07 (18)
C4A—N2A—C3A—C2A	−2.9 (3)	C4B—N2B—C3B—C2B	2.5 (3)
C5A—C2A—C3A—N5A	179.67 (19)	C5B—C2B—C3B—N5B	−179.83 (19)
N1A—C2A—C3A—N5A	1.9 (3)	N1B—C2B—C3B—N5B	−2.9 (3)
C5A—C2A—C3A—N2A	1.2 (3)	C5B—C2B—C3B—N2B	−0.4 (3)
N1A—C2A—C3A—N2A	−176.57 (19)	N1B—C2B—C3B—N2B	176.56 (18)
C3A—N2A—C4A—N3A	2.1 (3)	C3B—N2B—C4B—N3B	−2.3 (3)
C3A—N2A—C4A—N4A	−176.13 (18)	C3B—N2B—C4B—N4B	176.55 (17)
C5A—N3A—C4A—N2A	0.5 (3)	C5B—N3B—C4B—N2B	0.0 (3)
C5A—N3A—C4A—N4A	178.75 (18)	C5B—N3B—C4B—N4B	−178.80 (18)
C6A—N4A—C4A—N2A	172.1 (2)	C6B—N4B—C4B—N2B	−172.0 (2)
C6A—N4A—C4A—N3A	−6.3 (3)	C6B—N4B—C4B—N3B	6.9 (3)
C4A—N3A—C5A—O2A	177.46 (19)	C4B—N3B—C5B—O2B	−178.21 (19)
C4A—N3A—C5A—C2A	−2.3 (3)	C4B—N3B—C5B—C2B	2.0 (3)
C3A—C2A—C5A—O2A	−178.3 (2)	C3B—C2B—C5B—O2B	178.5 (2)
N1A—C2A—C5A—O2A	−0.5 (3)	N1B—C2B—C5B—O2B	1.4 (3)
C3A—C2A—C5A—N3A	1.4 (3)	C3B—C2B—C5B—N3B	−1.8 (3)
N1A—C2A—C5A—N3A	179.19 (17)	N1B—C2B—C5B—N3B	−178.80 (17)
C4A—N4A—C6A—O3A	12.3 (3)	C4B—N4B—C6B—O3B	−12.1 (3)
C4A—N4A—C6A—C7A	−166.52 (19)	C4B—N4B—C6B—C7B	167.13 (19)
O3A—C6A—C7A—C8A	−1.9 (3)	O3B—C6B—C7B—C8B	2.4 (3)
N4A—C6A—C7A—C8A	176.91 (19)	N4B—C6B—C7B—C8B	−176.8 (2)
O3A—C6A—C7A—C10A	−121.5 (2)	O3B—C6B—C7B—C10B	122.1 (2)
N4A—C6A—C7A—C10A	57.3 (2)	N4B—C6B—C7B—C10B	−57.1 (2)
O3A—C6A—C7A—C9A	117.2 (2)	O3B—C6B—C7B—C9B	−116.9 (2)
N4A—C6A—C7A—C9A	−64.0 (2)	N4B—C6B—C7B—C9B	63.9 (2)
O1A—C1A—C11A—C14A	−173.80 (19)	O1B—C1B—C11B—C14B	175.6 (2)
N1A—C1A—C11A—C14A	8.7 (3)	N1B—C1B—C11B—C14B	−6.4 (3)
O1A—C1A—C11A—C13A	−51.3 (3)	O1B—C1B—C11B—C13B	53.3 (3)
N1A—C1A—C11A—C13A	131.2 (2)	N1B—C1B—C11B—C13B	−128.7 (2)
O1A—C1A—C11A—C12A	66.4 (3)	O1B—C1B—C11B—C12B	−64.3 (2)
N1A—C1A—C11A—C12A	−111.1 (2)	N1B—C1B—C11B—C12B	113.8 (2)

### Hydrogen-bond geometry (Å, °)

**Table:**

*D*—H···*A*	*D*—H	H···*A*	*D*···*A*	*D*—H···*A*
N4A—H4AA···O1W^i^	0.86	2.07	2.918 (2)	167
N4B—H4BA···O4W^ii^	0.86	2.08	2.920 (2)	166
N5B—H5BA···O4W^iii^	0.86	2.32	3.160 (2)	166
N5B—H5BB···O1A^iv^	0.86	2.09	2.861 (2)	149
O1W—H1W1···O2W^v^	0.87	2.00	2.857 (2)	167
O1W—H2W1···O2W^vi^	0.90	1.92	2.819 (2)	178
O2W—H2W2···O2B^v^	0.89	1.96	2.824 (2)	162
O3W—H1W3···O2A^iii^	0.86	1.91	2.722 (2)	158
O3W—H2W3···O2A^vii^	0.89	1.97	2.833 (2)	162
O4W—H1W4···O3W^viii^	0.88	1.99	2.865 (2)	174
N3A—H3AA···O3A	0.86	1.98	2.633 (2)	132
N5A—H5AA···O1W	0.86	2.32	3.163 (2)	167
N5A—H5AB···O1B	0.86	2.08	2.854 (2)	149
N3B—H3BA···O3B	0.86	1.97	2.632 (2)	132
O2W—H1W2···O2B	0.87	1.91	2.717 (2)	154
O4W—H2W4···O3W	0.88	1.93	2.811 (2)	173
C14A—H14A···O1B	0.96	2.53	3.355 (3)	144

Symmetry codes: (i) −*x*+2, −*y*, −*z*+1; (ii) *x*, *y*−1, *z*; (iii) −*x*+1, −*y*+1, −*z*; (iv) *x*−1, *y*, *z*; (v) −*x*+1, −*y*, −*z*+1; (vi) *x*+1, *y*, *z*; (vii) *x*−1, *y*+1, *z*; (viii) −*x*+1, −*y*+2, −*z*.
